# A Pan-Cancer Comparative Analysis of The Cancer Genome Atlas Transcriptomic TIL-Immune Signatures

**DOI:** 10.21203/rs.3.rs-6441170/v1

**Published:** 2025-04-25

**Authors:** Kyle Hitscherich, Darryl Noussome, Aaron Dinerman, Victoria Dulemba, Frank Lowery, Naris Nilubol

**Affiliations:** National Cancer Institute; National Cancer Institute; National Cancer Institute; National Cancer Institute; National Cancer Institute; National Cancer Institute

**Keywords:** Cancer, Immunotherapy, Single Cell Sequencing, Tumor Microenvironment, Tumor Infiltrating Lymphocytes

## Abstract

Efforts to understand the tumor microenvironment (TME) through basic science research and The Cancer Genome Atlas (TCGA) data analysis have led to the creation of unique immune transcriptomic signatures from tumor-infiltrating lymphocytes (TIL). However, no pan-cancer analysis has been conducted to compare the prognostic performance of these signatures using overall survival (OS) or progression-free interval (PFI) as endpoints. We compiled a library of 146 TIL-immune signatures and evaluated gene signature score correlation with OS and PFI for 9,961 available TCGA samples across 33 cancer types. Zhang CD8 TCS demonstrated higher accuracy in prognosticating both OS and PFI across the pan-cancer landscape, however, variability was seen across cancer types and germ cell origin. Cluster analysis compiled a group of six signatures (Oh.Cd8.MAIT, Grog.8KLRB1, Oh.TIL_CD4.GZMK, Grog.CD4.TCF7, Oh.CD8.RPL, Grog.CD4.RPL32) whose association with OS and PFI could potentially be conserved across multiple cancer types.

## Introduction

The Cancer Genome Atlas (TCGA) program curated multi-omic data, clinical characteristics, and outcomes of over 10,000 primary cancers from 33 cancer types over the past 17 years.^1^ The goal of the project evolved from understanding the genetics of a select cancer histology, (glioblastoma multiforme) to now using combined tumor and microenvironment transcriptomics to better understand cancer biology as it relates to outcomes.^1^

Immunotherapy has evolved over the past two decades as a promising arm of cancer therapy that can provide robust and durable responses for a wide variety of cancer types.^2^ As success with checkpoint inhibitors (anti-PD-1, -PD-L1, -CTLA-4) and adoptive cell transfer (ACT, including chimeric antigen receptor (CAR) and tumor-infiltrating lymphocyte (TIL)) become more prevalent, the interest in the regulation of immune system in cancer microenvironment has grown.^3, 4, 5, 6, 7^ Multiple immune gene signatures have been developed from transcriptomic data looking to better describe TIL populations and how they may impact the tumor microenvironment (TME).^8, 9, 10, 11^ Several of these in-depth evaluations of the tumor-immune microenvironment have sought to identify populations of T-cells that may be associated with disease progression through cytotoxic function, neoantigen recognition, or more “stem-like” phenotypic state in specific cancers.^7^

In querying the TCGA, numerous research teams have sought to incorporate patient outcomes data into the development of such predictive gene signatures.^12, 13, 14, 15, 16 17^ In many instances, these signatures include similar genes compared to those constructed from direct primary patient source data, however, coupling outcomes data from TCGA database has allowed for the investigation of novel genes, some specific to rare cancers and their subtypes.^18, 19^

Although there were over 150 TIL-TIL-immune signatures published, no study has compared the prognostic performance of these TIL-immune signatures to identify the top-ranked predictive TIL-immune signatures across all cancer, individual cancer types, and germ-cell origins. Previous work has demonstrated utility in such signatures in predicting response rates to immunotherapy such as checkpoint inhibitors.^13, 14^ Further understanding of the ideal immune cell population found within TIL could expand such therapeutic tools and aid clinicians in selecting patients who may benefit from such therapies.

## Methods

### Gene signature library construction and sample accrual

A library of Tumor Infiltrating Lymphocyte (TIL) immune transcriptomic signatures was generated by PubMed literature review, specifically focusing on recent publications analyzing RNA-sequencing data on TIL derived from patients with metastatic cancers without restriction placed on histology. Emphasis was placed on publications identifying one or multiple T cell populations characterized by defined TIL-TIL-immune signatures (eg. “stem-like”, “terminally differentiated”, “effector memory”, “tissue resident memory” etc.). This library was broadened by searching “TCGA”, “transcriptomic” and “signature” to accumulate publications compiled from RNA-sequencing signatures specifically derived from TCGA database review, compared to those previously identified from direct, patient-sourced studies of immune transcriptomics and phenotypes. It should be noted that although some of these signatures were constructed for prognostic purposes, many were developed as a descriptive effort to define TIL populations within the TME. These TIL-TIL-immune signatures were then individually queried for all upregulated genes within their composition. Signature gene lists were reviewed to ensure consistent nomenclature across publications. Duplicated signatures were excluded from the final analysis.

The TCGA recount3 project is an online data source containing the accumulated RNA-sequencing data contained within the TCGA database and across 8,679 studies of human samples.^20^ RNA-sequencing data were downloaded for 33 cancer types and 9,961 samples. The recount3 project processed all RNA-seq samples via the Monorail system and provided gene-level counts using Gencode v26 (Table 1). The GSVA R/ Bioconductor package was used to calculate individual level gene set enrichment scores for each sample. Overall survival (OS) and progression-free interval (PFI) were chosen as primary endpoints similar to the previous publication by Liu et al.^1^ Based on the nature of TCGA database comprised of non-metastatic primary tumor lesions, we determined PFI as a potentially insightful metric for our study.

### Gene signature analysis and construction of a novel signature

OS and PFI coefficients were calculated based on the applicability of each TIL-TIL-immune signature for individual samples based on grouped populations. Our analysis was conducted across all cancer specimens, distinction by tissue germ cell origins, individual cancer type, and descriptive immune clusters.^21^ The association of each TIL-immune signature score and OS and PFI were compared. Analysis was performed for the above-mentioned populations to assess the performance of signatures and broad applicability.

Conserved genes found across multiple high-performing TIL-TIL-immune signatures were compiled. The top frequently conserved genes were used to construct a novel signature (Novel_Sig) and compared against the performance of those originally identified within our constructed library.

### Cluster analysis and concordance evaluation

Cluster analysis was performed on all signatures based on the genetic composition of each signature. The organization of clusters was determined to be 10 based on differentiation of positively and negatively related OS and PFI outcomes based on cluster association. Prognostication capability was evaluated for each cluster based on randomly sampled specimens via TCGA data. The remaining 10% of samples not included in prognostication were then used for Kaplan-Meyer analysis across constructed clusters.

## Results

### Construction of a gene signature library

**Figure 1:**
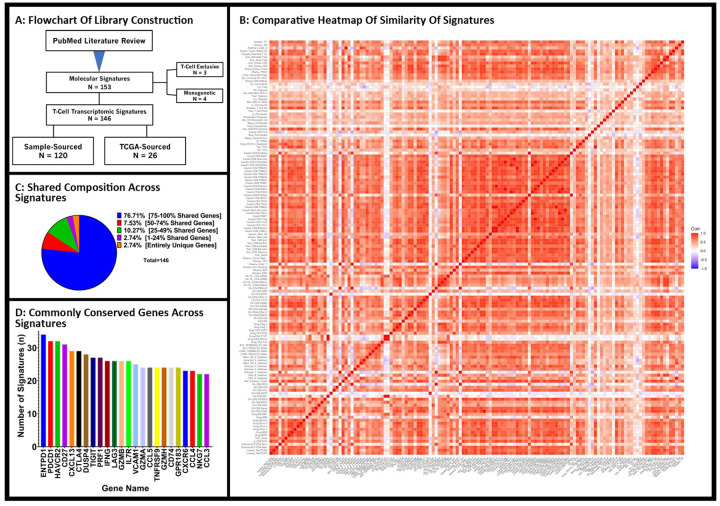
Construction of RNA-sequencing TIL-Immune Signature Library Reveals High Conservation of Composite Genes Across Signatures: Flowchart outlining the construction of our RNA-sequencing TIL-immune signature library with the inclusion of both primary samples sourced molecular signatures as well as TCGA-sourced samples (A). Comparing all 146 gene signatures amongst one another, we see a high level of similarity between signatures demonstrated via heatmap (B). Pie chart demonstrating the high level of shared genes comprising the majority of these compiled signatures (C). The most commonly shared genes include those commonly associated with more “exhausted” phenotypes (ENTPD1, PDCD1, HAVCR2, etc.) (D).

We identified 153 immune transcriptomic signatures from the literature review. Three were not included as they were not specific to T cells, and four were excluded as they only contained 1 gene. We thus examined 146 signatures across 45 publications (Fig. 1A). One hundred and twenty (120) signatures were described in the setting of basic science research and derived from single-cell TIL sequencing sourced directly from patient samples. Twenty-six (26) were developed from the review of available TCGA database and/or in combination with other databases such as the Gene Expression Omnibus (GEO) database. These signatures comprised 3088 unique genes with nearly half (1432) shared across multiple signatures (Supplemental Table 1). The average number of sourced samples for the development of a molecular signature was 132.7 with a median of 10 patient samples used. Most sourcing was restricted to a single cancer type (77%, 112/146) with the dominant cancer types including non-small cell lung cancer (NSCLC, 23%, 32/146), bladder cancer (20%, 29/146), and melanoma (20%, 29/146).

Examining the content of each signature, 1432 of the 3088 genes (46.37%) were shared between at least 2 signatures (Fig. 1B). Of these genes shared among multiple signatures, they were found in an average of 4.97 signatures with 65.5% shared across more than 3 signatures. More than 3 quarters (76.71%) of signatures contained 75–100% of genes shared amongst at least 2 signatures from our library (Fig. 1C). Only 2.74% of compiled signatures were comprised of entirely unique genes (Fig. 1C). The most overlapped genes across signatures were ENTPD1 (n = 34), PDCD1 (n = 32), and HAVCR2 (n = 32) (Fig. 1D). The median number of genes per signature was 50 with a range of 2 to 1114 genes (Supplemental table 1). From querying TCGA database we accumulated bulk RNA transcriptomic data from 9,961 patient samples across 33 tumor types (Table 1) (Supplemental Table 2).

### Examining performance across pan-cancer

**Figure 2:**
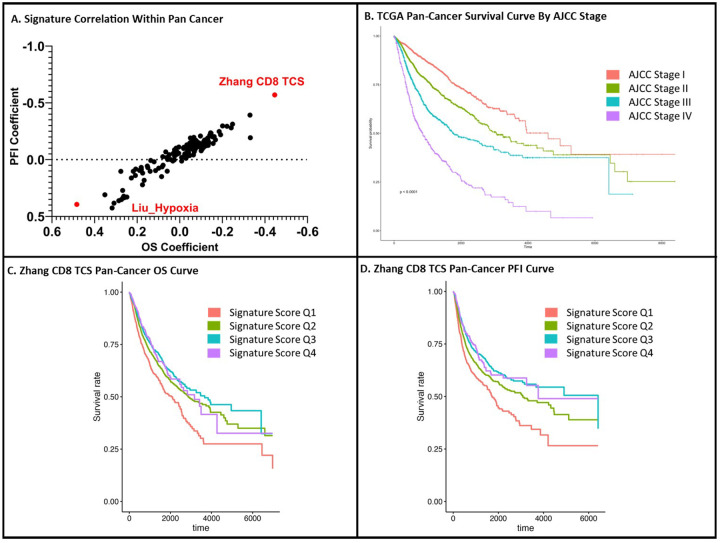
TIL-Immune Signature Performance Across Pan-Cancer Reveals Prognostic Capabilities for OS and PFI: Across our pan-cancer analysis, one signature (Zhang CD8 TCS) was associated with the longest OS and PFI across samples (A). When accounting for AJCC cancer staging, we were able to plot OS survival for patients demonstrated via the Kaplan-Meier curve (B). Stratifying samples instead by Zhang CD8 TCS score into quartiles, we see a distinction in OS and PFI correlating with higher signature scores (C and D).

To examine performance across pan-cancer we required grouping of samples to undergo gene signature score analysis due to the large number of available primary tumor samples. We opted to cluster samples by similarities in gene expression to forego any other grouping metric in an unbiased fashion. In doing so, we were able to evaluate gene signature scores as they related to OS and PFI coefficients of similar transcriptomic cancer samples (Supplemental Fig. 1A). Across our 146 gene signatures, the Zhang CD8 T-Cell associated gene signature for prognosis risk in lung adenocarcinoma (Zhang CD8 TCS) appeared to have the lowest OS and PFI coefficients, consistent with the strongest association with longer OS and PFI (Fig. 2A, Supplemental Table 3).^16^ Alternatively, the Liu hypoxia-associated gene score in bladder cancer (Liu_Hypoxia) appeared to have the highest OS and PFI coefficients, consistent with an association with shorter OS and PFI (Fig. 2A).^1^

Within our pan-cancer analysis, patient OS correlated with the American Joint Committee on Cancer (AJCC) staging, as expected (Fig. 2B). Scoring samples based on Zhang CD8 TCS gene expression, patient samples were separated into quartiles based on concordance with molecular signature expression. When comparing OS from Q2 to Q1 we found a hazards ratio of 0.74 (P = 9.95e-^7^) consistent with significantly longer OS with higher concordance of Zhang CD8 TCS gene signature. Similar significance was found comparing Q3 (HR = 0.67, P = 2.39e-^9^) and Q4 (HR = 0.68, P = 1.72e-^4^) (Fig. 2C). Thus the higher correlation quartiles (Q2-Q4) were associated with prolonged OS and PFI compared to the lowest sample quartile (Q1) (Fig. 2C and 2D).

### Examining performance across germ cell origin

**Figure 3:**
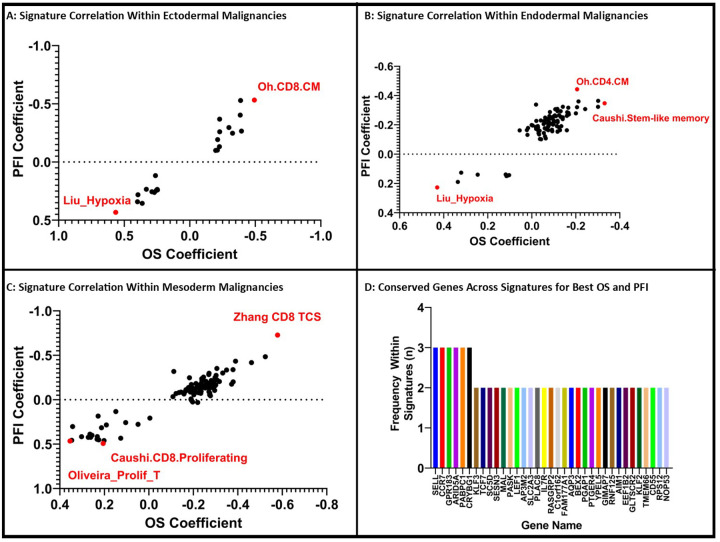
TIL-Immune Signature Performance Differentiates Across Germ Cell Origin Samples: By grouping samples based on germ cell origin, we identify several signatures whose scores correlated with OS and PFI across Ectoderm (A), Endoderm (B) and Mesoderm (C) malignancies. There were several conserved genes found across several of these top-performing signatures (D).

An alternative grouping of samples was performed by germ-cell origin rather than overall transcriptomic similarities. This offered additional insight into the performance of our 146 signatures (Fig. 3A–C). Although many signatures did not demonstrate a statistically significant correlation between OS or PFI coefficients and signature score, several signatures began to show a direct or inverse association with OS and/or PFI coefficients (Supplemental Table 4).

When examining signature score by germ cell origin, the Oh CD8 central memory signature (Oh.CD8.CM) and Zhang.CD8.TCS had the highest correlations with OS and PFI coefficients for ectoderm and mesoderm-derived cancers, respectively (Fig. 3A and 3C).^9, 16, 22^ In endoderm-derived malignancies, the top signatures correlating with OS and PFI coefficient were the Caushi Stem-like memory T-cell signature (Caushi.Stem-Like memory) and Oh CD4 central memory signature (Oh.CD4.CM) respectively (Fig. 3B).^9, 22^ Thus, patients whose primary tumors contained more T-cells corresponding with these gene signatures tended towards longer OS and PFI following resection (Table 2). On closer examination, we identified 21.5% of composite genes within these signatures (n = 35/163) were shared across multiple signatures (Supplemental Table 5). The most conserved genes across these signatures included GPR183, CCR7, SELL, ARID5A, and others (Fig. 2D).

We conversely identified that several signatures had an inverse correlation with OS and PFI coefficients. The signature titled Liu_Hypoxia had the poorest prognostication for Ectoderm and Endoderm derived malignancies (Figs. 3A and 3B).^23^ The Oliveira proliferating T-cell gene signature (Oliveira_Prolif_T) and Caushi proliferating CD8 T-cell signature (Caushi.CD8.Proliferating) correlated with shorter PFI and OS respectively within the mesoderm-derived malignancies (Fig. 3C).^9, 10^ Of these TIL-immune signatures, 10.6% (n = 18/170) of their genes were shared amongst two of these signatures, however, no gene was shared across all 3 signatures associated with poor OS and PFI.

### Examining performance across cancer types

By distinguishing individual cancer types, rather than germ cell origin, we saw greater variability across OS and PFI coefficients as correlates to gene signature score (Supplemental Table 6). We determined that across our 33 cancer types, 22 had statistically significant variability across the signature score to correlate with OS coefficient and 28 regarding PFI coefficient (Tables 3 and 4).

**Figure 4:**
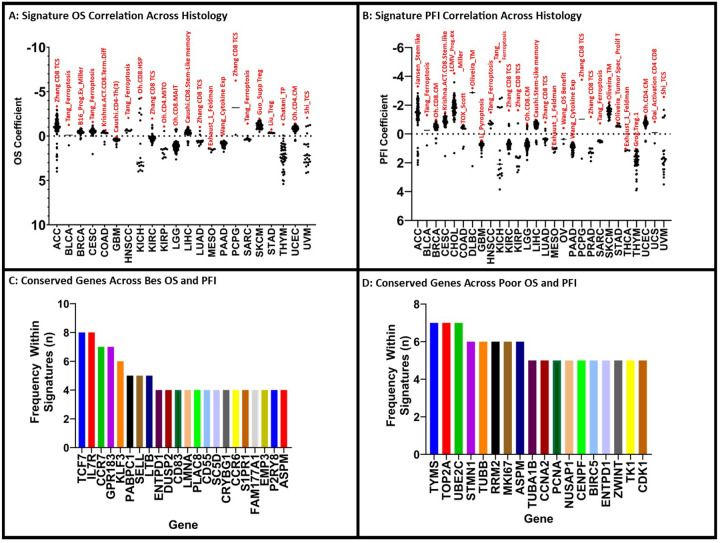
TIL-Immune Signature Performance Differentiates Across Individual Cancer Histology: By grouping samples based on cancer histology, we demonstrate several signatures whose score correlates with improved OS (A) and PFI (B). Across these top-performing signatures, numerous genes are shared (C). Similarly, for poorly performing signatures numerous genes are shared (D).

Examining the correlation between immune gene signature and OS showed variability in performance across different cancer types. Across the 22 cancers, 16 unique signatures were found to have the highest correlation to OS coefficient. Despite this variability, two signatures were found to correlate with OS coefficient across multiple cancer types, the Tang ferroptosis related gene signature in head and neck squamous cell carcinoma (Tang_Ferroptosis) and Zhang.CD8.TCS, each seen across 4 cancer types (BLCA, CESC, HNSC, SARC and ACC, KIRC, LUAD, PCPG) (Fig. 4A, Table 3).^15, 16^

Regarding the PFI coefficient, 17 signatures were found to be correlated with longer PFI across our 28 cancer types. Similar to OS, Tang_Ferroptosis and Zhang.CD8.TCS signatures shared high correlation with PFI. The Zhang.CD8.TCS signature was the best correlate to PFI within five cancer types (KIRC, KIRP, LUAD, PCPG, PRAD) and Tang_Ferroptosis in four (BLCA, HNSC, KICH, SARC) (Fig. 4B, Table 4).^15, 16^ The number of shared genes across top correlates for cancer type was 20.2% (n = 223/1106). The most common overlapped genes across these signatures were TCF7, IL7R, CCR7, GPR183, KLF3, PABPC1, SELL and LTB (Fig. 4C).

An inverse correlation with multiple TIL-immune signatures across different types of cancer was also identified. Signatures Caushi.CD8.Proliferating and the An immune cell prognosticating signature in cervical cancer (AnCervicalCA) were most frequently found to be inversely correlated to OS across multiple cancer types, both for 3 different cancer types (ACC, MESO, KIRP and KICH, LIHC, SARC) (Supplemental Table 6).^9, 24^ Looking at PFI, Caushi.CD8.Proliferating was most frequently the top inverse correlation found across 6 cancer types (ACC, LIHC MESO, SARC, KIRC, KIRP) (Supplemental Table 6).^9^ When we examined the gene composition of these gene signatures (Caushi.CD8.Proliferating, AnCervical CA, Duhen_Tumor React CD8, Liu_Hypoxia, Chatani_TP, Tang_Ferroptosis, LCMV_PROG.EX_Miller, Qi_TREG, Li_Pyroptosis, Jansen_Term diff, Ahuluwalia_Prognostic Cell Death, Wu_OS Pancreatic CA, Yan_TCS, Exhaust_1_Feldman, Caushi.CD4-Th(3), Grog.8TRM.2, Hou_T Cell Prolif, Krishna.ACT.CD8.Term.Diff, Oh.CD4.PROLIF, Oh.CD8.MITO, Oliveira_AAT, Oliveira_Prolif_T, Oliveira_Tumor Spec_Prolif T, Yang_Cupropptosis), we found that 23.74% of their genes were shared across multiple signatures (n = 151/636). The most conserved genes across these signatures were TYMS, TOP2A, and UBE2C each found within 28% of signatures (n = 7/25) (Fig. 4D).

### Examining performance across immune cell clusters

We next attempted to analyze gene signature performance by grouping samples by immune cells clustered based on likely phenotypic state. These groups included such categories as wound healing, IFN-gamma dominant, Inflammatory, Lymphocyte-depleted, immunologically quiet and TGF-B dominant.^21^ Our analysis, however, did not demonstrate a statistically significant correlation between gene signature score and OS or PFI coefficients.

### Cluster analysis of TIL-Immune Signatures

**Figure 5:**
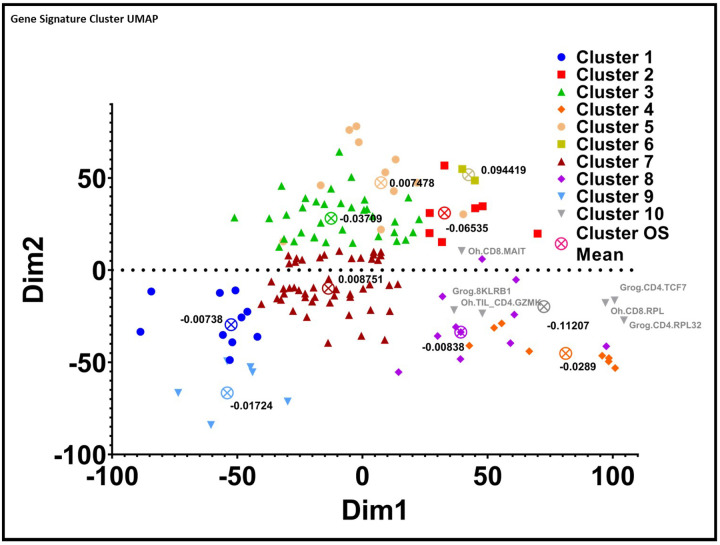
Cluster Analysis of Signatures Identifies High Performing Similar Signatures: By clustering signatures based on the similarity of their composite genes, we can analyze cluster performance against pan-cancer samples. The mean OS coefficient is plotted for each cluster.

Mapping our TIL-immune signature library by composite genes allowed us to cluster similar signatures into groups. We opted to cluster all signatures into a total of 10 groups as this was the minimal cluster number where the distinction for both correlation and inverse correlation for OS and PFI could be detected (Fig. 5, Table 5, Supplemental Fig. 1B). Cluster sizes ranged from 2 to 48 signatures per cluster with a median size of 9 signatures (Supplemental Table 7). By examining group prognostication of OS and PFI we were able to extrapolate that cluster 10 was associated with the longest mean OS and PFI across pan-cancer samples (Table 5). The mean OS and PFI coefficients were − 0.11207 and − 0.16673 respectively.

### Constructing a novel gene signature

By examining the top gene signatures correlating with both OS and PFI across our distinctions of germ cell origin and cancer tumor type, we found that there were 28 unique gene signatures in total that were the highest correlates to OS or PFI within a given category.^8–10, 12, 15, 16, 18, 22–23, 25–34^. We extracted the top 22 genes shared across these signatures including IL7R, TCF6 (seen in 8 signatures), CCR7 and GPR183 (Seen in 7 signatures), etc. (Table 6, Supplemental Table 8).

Examining our novel signature (Novel_Sig) showed concurrence with improved OS (OS coefficient − 0.072) and PFI (PFI coefficient (−0.126 ) compared to the mean across all signatures (OS coefficient: −0.016, PFI coefficient: −0.059) within pan-cancer (Supplemental Table 9). However, our Novel_Sig was not within the top 50 signatures correlated with either prolonged OS or PFI. When examining performance within germ cell origin, our Novel_Sig signature was associated with an OS coefficient less than the average for all signatures within Mesoderm (−0.445 vs. −0.349) and Endoderm (−0.125 vs 0.014) derived cancers (Supplemental Table 10). Regarding PFI, our Novel_Sig was only associated with a PFI coefficient less than the average of all signatures within Endoderm derived cancers (−0.146 vs. 0.039) (Supplemental Table 10). Within both OS and PFI, our Novel_Sig was not within the top correlates, or inverse correlates for OS or PFI across all germ cell origins (supplemental Table 10).

When examining tumor types, our Novel_Sig signature only had a statistically significant association with OS or PFI coefficient in 6 cancer types (BRCA, CESC, CHOL, LGG, LIHC, PAAD). Considering OS, our Novel_Sig had an OS coefficient less than the mean across all other signatures within CESC (−0.697 vs. −0.520), CHOL (−0.946 vs. −0.919), LIHC (−0.532 vs. −0.375) and PAAD (0.573 vs. 0.934) (Supplemental Table 11). Only within PAAD did our Novel_Sig score amongst the top 5 signatures with the lowest OS coefficient, otherwise our signature was only modestly below the mean for OS coefficients (Supplemental Table 11). Regarding PFI our Novel_Sig had a PFI coefficient below the mean of all other signatures within BRCA (−0.512 vs. −0.492), CESC (−1.043 vs. −0.089), CHOL (−2.035 vs. −1.878), LIHC (−0.737 vs. −0.538) and PAAD (0.696 vs. 0.99), however in none of these cancer types was our signature within the top lowest PFI coefficients (Supplemental Table 11).

## Discussion

Given the availability and accessibility of genome-wide high-throughput transcriptomic data in cancers, numerous TIL-immune signatures were created and used in bulk transcriptomic data to aid prognostication and predict treatment response to various immunotherapy regimens. In addition, these TIL-immune signatures can improve our understanding of the presence of different immune cells in the cancer microenvironment and the associations with clinical outcomes in the absence of single nuclei transcriptomics performed in large cohorts. However, there were systemic and direct comparisons to assess the differences (or similarities) between these TIL-immune signatures to understand their roles in prognostication across cancer types. To address this gap of knowledge, we curated and compared the components and the performance in prognostication of 146 published TIL-immune signatures in all cancers in TCGA database. Reviewing signature performance across pan-cancer samples, we showed that the Zhang CD8 TCS signature had the overall closest correlation with patient OS and PFI.^16^ Survival curves for both OS and PFI demonstrated statistically significant prolonged OS and PFI in TCGA samples demonstrating higher concordance with this Zhang CD8 TCS score (Fig. 2). A more nuanced investigation into the performance of these signatures, however, realizes the disparity in signature performance across cancer types. Through our germ cell origin analysis, we see that the Zhang CD8 TCS signature was the most proficient signature correlate of OS and PFI in mesoderm-derived malignancies, however in ectoderm and endoderm-derived malignancies, other signatures (Oh.CD8.CM, Oh.CD4.CM, Caushi-Stem like memory) were associated with lower OS and PFI coefficients. The performance of each TIL-immune signature in predicting OS and PFI varied across different cancer types. Although Zhang CD8 TCS had the lowest OS coefficient across ACC, KIRC, LUAD, PCPG and lowest PFI coefficient in KIRC, KIRP, LUAD, PCPG, and PRAD there is much more variability in top-performing signatures such as the Miller progenitor exhausted T-cell signature (B16_prog.Ex_miller), the Guo suppressive CD4 T-regulatory cell signature (Guo_Supp Treg) and the Chatani CD8 tumor recognition signature (Chatani_TP) having the lowest OS coefficients in BRCA, SKCM, and THYM respectively. In many instances, one-off signatures not seen as top correlates to OS or PFI across pan-cancer or germ cell origin are top predictors of outcomes for individual cancer types. Reliance on the Zhang CD8 TCS signature for all cancers as a prognostic indicator for OS and PFI could provide valuable insight into future cancer behavior, however, our study demonstrates the importance of consideration of numerous published signatures tailored to tumor type and origin to better inform patient-centered decision-making.

It should be noted that within our studies of interest, there was variability in the cancer types by which their signatures were derived. Most publications utilized samples from a single histology to generate descriptive signatures (77% of signatures, 112/146) (Supplemental Table 12). Only 6 publications with 34 resultant signatures (23%, 34/146) utilized multiple cancer types to generate TIL-immune signatures.^7, 14, 29, 35–37^ Of studies utilizing a single cancer histology, the most common included non-small cell lung cancer (NSCLC, 23%, 32/146), bladder cancer (20%, 29/146), and melanoma (20%, 29/146). (Supplemental Table 12). It is no coincidence that these cancer types represent the most “immune-friendly” solid tumors, with checkpoint blockade therapy FDA-approved for all three, including in the management of adjuvant therapy after primary tumor resection.^38–40^ The emerging role of neoadjuvant checkpoint blockade in the management of these tumors creates a scientific dilemma as checkpoint blockade has an unclarified role in altering T-cell phenotype. Despite the small number of studies utilizing numerous cancer types, we did identify multiple TIL-immune signatures that were top prognostic indicators across multiple cancers (Tang_Ferroptosis and Zhang.CD8.TCS) (Fig. 4A, Supplemental Table 6). Both were derived from a single histology, HNSC and LUAD respectively. Unsurprisingly, they were also top prognosticators of OS and PFI within their derivative cancer types. Regardless, these two signatures and two others, the Li T-cell pyroptosis signature in glioblastoma (Li-Pyroptosis) and the Oliveira T-cell memory gene signature (Oliveira_TM) outperformed gene signatures derived from the same histology they were prognosticating. Additionally, only 1 signature derived from multiple cancer types was found to correlate highly with PFI or OS. This signature, the Grog T regulatory CD4 signature (Grog.Treg.1), did not correlate with higher performance within any of the cancer types it was derived from (Fig. 4A and 4B, Supplemental Table 6).^29^ It follows that the immune landscape amongst differing cancer types is likely more complex than anticipated.

Signature composition across our library revealed that despite different goals in developing signatures, many genes were conserved across multiple signatures. Notably, many of these conserved genes (ENTPD1, PDCD1, HAVCR2, CXCL13) are those most frequently associated with T cell exhaustion.^7, 11, 31^ At the onset of our investigation, we surmised that evidence of tumor recognition, as exemplified by increasing signatures of T-cell exhaustion, would drive immune response and lead to improved overall prognosis (Hanada 2022, Lowery 2022, Duhen 2022, Duhen 2018, Chatani 2023).^7, 11, 26, 36–37^ However, only two signatures describing neoantigen reactivity (Chatani_TP, Oliveira_Tumor Spec_Prolif T) were found to have correlations with OS or PFI within histology or germ cell origin analysis.^10, 26^ Likely, neoantigen recognition alone is not enough to prognosticate tumors as phenotypic state plays a role in describing the overall immune response to cancer.^31^

Although the original hypothesis of tumor recognition did not seem apparent, our data did frequently identify signatures expressing genes associated with a “less exhausted” cell phenotype as top prognosticators of OS and PFI (Caushi.stem-like memory, Caushi.CD8.Stem-like memory, B16_Prog.Ex_Miller, Jansen_Stem like, Krishna.ACT.CD8.Stem.Like, LCMV_Prog.EX_Miller).^9, 25, 28, 31^ Many of these signatures feature genes associated with more “stem-like” phenotype (IL7R, TCF7, SELL, CCR7, etc) (Table 6). Despite this association, our novel signature, comprised of these conserved genes, had middling performance across pan-cancer, germ cell, and individual cancer types. Much like neoantigen recognition, “stem-like” phenotype alone is not indicative of better prognostication amongst primary cancers.

Across our multiple iterations of signature library analysis, Zhang CD8 TCS was the most consistent prognosticator of OS and PFI. This signature was the top prognosticator across multiple cancer types, one germ cell origin category, and across our pan-cancer analysis as well. The signature was initially constructed through an analysis of available RNA sequencing data for LUAD from TCGA with the intention of constructing a signature of CD8 markers that could predict likely response to immune checkpoint therapy in LUAD patients.^16^ The signature itself consists of multiple genes describing T-cell adhesion, early activation, cytokine receptors, and aquaporins. No particular indication of the T-cell phenotypic state was considered when constructing this signature, again lending credence to the idea that the TME and immune cell phenotypic environment is much more complex than anticipated. That said, we believe that use of the Zhang CD8 TCS score could be utilized in patient counseling following surgical resection, or possibly even in the decision algorithm for receipt of adjuvant therapy. Consideration for other well-performing signatures based on cancer cell origin and cancer type should also be considered if mRNA sequencing data is available to potentially assist in prognosticating cancer behavior and the immunoregulatory response following resection of primary tumors.

Despite promising potential, these conclusions require additional investigation and confirmation. Although TCGA does include useful metrics such as patient outcomes measures (OS, PFI, etc.), their capture of treatment modalities remains a barrier to more in-depth analysis. As was prevalent amongst many of the signatures we investigated, association of immune score with immune checkpoint blockade would be invaluable in selecting appropriate patients for therapy. Our study did not assess the association between these TIL-immune signatures and patient outcomes by the use of immune checkpoint blockade because the transcriptomic data in many cancers in TCGA were completed before the use of the first FDA-approved immune checkpoint blockade in 2011 became widely adopted. Further investigation into the application of metastatic cancer lesions could prove useful in guiding patients and practitioners in determining complex patient care strategies to combat advanced-stage cancers with systemic immune response activation.

In summary, the analysis across pan-cancer, germ cell origin, and individual histology revealed that the Zhang CD8 TCS signature demonstrated the best performance across the broadest scenarios in prognosticating OS and PFI for primary resected tumors. Numerous other signatures, however, perform well in OS and PFI performance when restricted to individual germ cell origin or within individual histology. Variability in prognostication could be due to numerous factors such as cancer behavior, histology, T-cell population, and phenotypic state. Further investigation is warranted to better understand the landscape of TIL populations and their potential in prognosticating and directing patient therapy.

## Figures and Tables

**Table 1:** TCGA tumor types, Nomenclature, and Available Samples for Analysis

Ectoderm/Neural Crest n = 2646 (26.6%)	
Head and Neck Squamous Cell Carcinoma (HNSC)	522 (5.3%)
Breast invasive Carcinoma (BRCA)	1,093 (11%)
Pheochromocytoma and Paraganglioma (PCPG)	179 (1.8%)
Skin Cutaneous melanoma (SKCM)	103 (1.0%)
Uveal Melanoma (UVM)	80 (0.8%)
Brain Lower Grade Glioma (LGG)	514 (5.2%)
Glioblastoma Multiforme (GBM)	155 (1.6%)
Mesoderm n = 3457 (34.7%)	
Mesothelioma (MESO)	87 (0.9%)
Sarcoma (SARC)	259 (2.7%)
Acute Myeloid Leukemia (LAML)	126 (1.3%)
Adrenocortical Carcinoma (ACC)	79 (0.8%)
Cervical Squamous Cell Carcinoma (CESC)	304 (3.2%)
Ectoderm/Neural Crest n = 2646 (26.6%)	
Kidney Chromophobe (KICH)	66 (0.7%)
Kidney Renal Clear Cell Carcinoma (KIRC)	531 (5.3%)
Kidney Renal Papillary Cell Carcinoma (KIRP)	290 (3.1%)
Uterine Corpus Endometrial Carcinoma (UCEC)	541 (5.4%)
Uterine Carcinosarcoma (UCS)	57 (0.6%)
Testicular Germ Cell Tumor (TGCT)	150 (1.5%)
Prostate Adenocarcinoma (PRAD)	497 (5.0%)
Lymphoid Neoplasm Diffuse Large B-Cell Lymphoma (DLBC)	48 (0.5%)
Ovarian Serous Cystadenocarcinoma (OV)	422 (4.2%)
Endoderm n = 3858 (38.7%)	
Thymoma (THYM)	120 (1.2%)
Bladder Urothelial Carcinoma (BLCA)	408 (74.5%)
Cholangiocarcinoma (CHOL)	36 (0.4%)
Colon Adenocarcinoma (COAD)	458
Ectoderm/Neural Crest n = 2646 (26.6%)	
	(4.6%)
Esophageal Carcinoma (ESCA)	184 (1.9%)
Liver Hepatocellular Carcinoma (LIHC)	371 (3.7%)
Lung Adenocarcinoma (LUAD)	516 (5.2%)
Lung Squamous Cell Carcinoma (LUSC)	501 (5.0%)
Thyroid Carcinoma (THCA)	505 (5.1%)
Stomach Adenocarcinoma (STAD)	415 (4.2%)
Rectum Adenocarcinoma (READ)	166 (1.7%)
Pancreatic Adenocarcinoma (PAAD)	178 (1.8%)

**Table 2:** Top TIL-immune signatures that correlated with overall survival (OS) and progression-free interval (PFI) by germ cell origin

Germ Cell Origin	Signature Title	OS Coefficient	OS Coefficient P Value	PFI Coefficient	PFI Coefficient P value
Neural Crest	Shi_TCS	−1.29137	0.001457	−1.66787	9.21E-6
Mesoderm	Yost_CD8_Memory	−0.66138	0.000233	−0.75684	5.8E-6
Ectoderm	Oh.CD8.CM	−0.61975	0.012588	−0.47997	0.029186
Endoderm	Zhang CD8 TCS	−0.60349	1.76E-10	−0.66531	1.61E-13
Mesoderm	Oh.CD4.CXCL13	−0.54667	0.017151	−0.98599	5.72E-6
Neural Crest	Oh.CD8.PRO	0.81151	0.005707	1.13123	6.13E-5

**Table 3:** Top TIL-immune signatures that correlated with overall survival (OS) by tumor type

Tumor Histology	Signature Title	OS Coef	OS Pvalue	PF Coef	PF Pvalue
PCPG	Zhang CD8 TCS	−6.24199	0.022149674	−3.76982	0.002143
KICH	Oh.CD8.HSP	−4.30571	0.008573393	−2.44199	0.042444
ACC	Zhang CD8 TCS	−3.34056	4.82561E-05	−2.38948	0.000287
UVM	Shi_TCS	−2.05644	0.003787896	−2.57423	7.04E-05
HNSC	Tang_Ferroptosis	−2.03379	3.02851E-10	−1.5004	6.04E-06
KIRC	Zhang CD8 TCS	−1.96574	4.80286E-10	−1.63795	3.9E-07
SKCM	Guo_Supp Treg	−1.96074	0.01343793	−1.98457	0.004273
CESC	Tang_Ferroptosis	−1.57918	0.003553691	−1.22583	0.024684
UCEC	Oh.CD4.CM	−1.40552	0.000572013	−1.27701	0.000207
SARC	Tang_Ferroptosis	−1.39883	0.00220507	−0.73848	0.047949
BLCA	Tang_Ferroptosis	−1.24705	0.000695187	−1.31438	0.000466
LIHC	Caushi.CD8.Stem-like memory	−1.0595	0.001002037	−0.91496	0.000695
LUAD	Zhang CD8 TCS	−1.01043	7.38238E-05	−0.81351	0.000407
BRCA	B16_PROG.EX_Miller	−0.91433	0.000962026	−0.84596	0.001901
LGG	Oh.CD8.MAIT	−0.81017	0.009074414	−0.66166	0.00686
STAD	Liu_Treg	−0.73121	0.000587008	−0.73239	0.001334
COAD	Krishna.ACT.CD8.Term.Diff	−0.5895	0.020006639	−0.55552	0.012663
KIRP	Oh.CD4.MITO	−0.55617	0.04930259	−0.7407	0.003681
GBM	Caushi.CD4-Th(3)	0.502069	0.046295118	0.735503	0.00316
PAAD	Wang_Cytokine Exp	0.503322	0.014469427	0.556764	0.003478
MESO	Exhaust_1_Feldman	1.38126	5.86199E-09	0.933397	0.000167
THYM	Chatani_TP	1.629962	0.042234695	1.211128	0.021425

**Table 4:** Top Progression Free Interval (PFI) Correlation for Each Histology

Tumor Histology	Signature Title	OS Coef	OS Pvalue	PF Coef	PF Pvalue
ACC	Jansen_Stem like	−2.89192	0.000666	−3.30626	6.13E-06
BLCA	Tang_Ferroptosis	−1.24705	0.000695	−1.31438	0.000466
BRCA	Oh.CD8.CM	−0.71512	0.040587	−0.99211	0.00404
CESC	Krishna.ACT.CD8.Stem.Like	−0.9932	0.012153	−1.50782	0.000245
CHOL	LCMV_PROG.EX_Miller	−1.82733	0.127838	−4.2128	0.002698
COAD	TOX_Scott	−0.66957	0.051828	−0.63124	0.038797
DLBC	Oliveira_TM	−1.13396	0.506353	−3.18516	0.029162
GBM	Li_Pyroptosis	0.083296	0.649199	0.445846	0.019261
HNSC	Tang_Ferroptosis	−2.03379	3.03E-10	−1.5004	6.04E-06
KICH	Tang_Ferroptosis	−4.19384	0.021295	−5.02205	0.004432
KIRC	Zhang CD8 TCS	−1.96574	4.8E-10	−1.63795	3.9E-07
KIRP	Zhang CD8 TCS	−1.07239	0.087701	−1.65042	0.003526
LGG	Oh.CD8.CM	−0.68851	0.038533	−0.72399	0.008386
LIHC	Caushi.Stem-like memory	−1.03655	0.001353	−0.9826	0.000267
LUAD	Zhang CD8 TCS	−1.01043	7.38E-05	−0.81351	0.000407
LUSC	Dai_Activation CD4 CD8	−0.10698	0.520351	−0.42427	0.03222
MESO	Exhaust_1_Feldman	1.38126	5.86E-09	0.933397	0.000167
OV	Wang_OS Benefit	−0.12112	0.57786	−0.41078	0.042113
PAAD	Wang_Cytokine Exp	0.503322	0.014469	0.556764	0.003478
PCPG	Zhang CD8 TCS	−6.24199	0.02215	−3.76982	0.002143
PRAD	Zhang CD8 TCS	−0.17488	0.892955	−1.18777	0.003254
READ	Ahuluwalia_Prognostic Cell Death	0.165135	0.840801	−1.43142	0.041254
SARC	Tang_Ferroptosis	−1.39883	0.002205	−0.73848	0.047949
SKCM	Oliveira_TM	−0.96642	0.240344	−2.22317	0.003376
STAD	Oliveira_Tumor Spec_Prolif T	−0.41472	0.081555	−0.77828	0.002654
THCA	Exhaust_1_Feldman	−0.11051	0.823879	1.022291	0.001125
THYM	Grog.Treg.1	0.805856	0.293964	1.044679	0.037388
UCEC	Oh.CD4.CM	−1.40552	0.000572	−1.27701	0.000207
UCS	Dai_Activation CD4 CD8	−0.564	0.147362	−0.84535	0.035912
UVM	Shi_TCS	−2.05644	0.003788	−2.57423	7.04E-05

**Table 5:** OS Coefficients for TIL-Immune Signatures Within Cluster #10

Signature Name	OS Coefficient
Grog.8KLRB1	−0.03943
Grog.CD4.RPL32	−0.44604
Grog.CD4.TCF7	−0.00901
Oh.CD8.MAIT	−0.08677
Oh.CD8.RPL	0.012838
Oh.TIL_CD4.GZMK	−0.10399

**Table 6:** Gene Composition of Novel Signature

Gene Name	Number of Signatures Present Within (n)
*IL7R*	8
*TCF7*	8
*CCR7*	7
*GPR183*	7
*KLF3*	6
*LTB*	5
*PABPC1*	5
*SELL*	5
*ASPM*	4
*CCR6*	4
*CD55*	4
*CD83*	4
*CRYBG1*	4
*DUSP2*	4
*EMP3*	4
*ENTPDq*	4
*FAM177A1*	4
*LMNA*	4
*P2RY8*	4
*PLAC8*	4
*S1PR1*	4
*SC5D*	4

## Data Availability

The data that support the findings of this study are available from the corresponding author, KJH, upon reasonable request. Data is otherwise publicly available within the TCGA database available in its initial publication at doi: 10.1016/j.cell.2018.03.022 or at their website https://www.cancer.gov/ccg/research/genome-sequencing/tcga. No additional data was generated in preparation of this manuscript.
